# CalPen (Calculator of Penetrance), a web-based tool to estimate penetrance in complex genetic disorders

**DOI:** 10.1371/journal.pone.0228156

**Published:** 2020-01-29

**Authors:** Aditya Addepalli, Sakhare Kalyani, Minali Singh, Debashree Bandyopadhyay, K. Naga Mohan

**Affiliations:** Department of Biological Sciences, Hyderabad, India; German Cancer Research Center (DKFZ), GERMANY

## Abstract

Mutations conferring susceptibility to complex disorders also occur in healthy individuals but at significantly lower frequencies than in patients, indicating that these mutations are not completely penetrant. Therefore, it is important to estimate the penetrance or the likelihood of developing a disease in presence of a mutation. Recently, a method to calculate penetrance and its credible intervals was developed on the basis of the Bayesian method and since been used in literature. However, in the present form, this approach demands programming skills for its utility. Here, we developed ‘CalPen’, a web-based tool for straightforward calculation of penetrance and its credible intervals by entering the number of mutations identified in controls and patients, and the number of patients and controls studied. For validation purposes, we show that CalPen-derived penetrance values are in good agreement with the published values. As further demonstration of its utility, we used schizophrenia as an example of complex disorder and estimated penetrance values for 15 different copy number variants (CNVs) reported in 39,059 patients and 55,084 controls, and 145 SNPs reported in 45,405 patients and 122,761 controls. CNVs showed an average penetrance of 7% with 22q11.21 CNVs having highest value (~20%) and 15q11.2 deletions with lowest value (~1.4%). Most SNPs, on the other hand showed a penetrance of 0.7% with rs1801028 having the highest penetrance (1.6%). In summary, CalPen is an accurate and user-friendly web-based tool useful in human genetic research to ascertain the ability of the mutation/ variant to cause a complex genetic disorder.

## Introduction

In contrast to the simple Mendelian disorders where mutation of a gene is always associated with the disease, in case of complex disorders, mutations in multiple genes are not always associated with the disease but contribute as risk factors. Therefore, the risk-conferring mutations are also found in normal controls. In this context, geneticists use statistics to determine first whether the mutation occurs at a significantly higher frequency in patients than in controls to establish an association. Once such significant association is established, it becomes important to determine penetrance (likelihood of causing the disease) of the mutation. Accordingly, a mutation can be either completely (100%) penetrant in which the presence of mutation definitively causes the disease or highly penetrant (there is more than 50% chance that the presence of mutation causes the disease) or low penetrant (there is less than 50% likelihood that the mutation causes the disease) [1]. However, in complex genetic disorders, the identified mutations are often incompletely (<100%) penetrant [2]. In order to calculate penetrance for a mutation conferring risk for a complex disease, Vassos et al [3] developed a Bayesian method, which is currently being used worldwide. According to this method, five types of data are needed: (i) the number of mutations identified in a patient sample, (ii) the number of patients studied, (iii) the number of mutations identified in the control sample, (iv) the number of controls studied and, (v) the general incidence of the disease under investigation in the population from which patients and controls are sampled (lifetime morbidity risk or baseline risk). This method involved simulation using the R statistical package to make prior distributions based on the observed frequencies in controls and patients. From these curves, 2.5^th^, 50^th^ and 97.5^th^ percentiles are extracted to obtain the median penetrance and its ~95% credible intervals. However, these calculations require programming skills which are generally not familiar to geneticists. Given these difficulties, a user-friendly web-based tool is desirable for direct determination of penetrance and its credible intervals without going through the need for programming skills. If such tool is available, then it would enable calculation of penetrance for mutations reported in a number of studies for common disorders and may eventually result in a database that will be useful in prenatal genetic screening and genetic counselling.

Here, we used python interface to develop ‘CalPen’, a free web-based tool, that enables calculation of penetrance and its ~95% credible intervals. We show that the values obtained using CalPen were in good agreement with the reported values. Using published data, we also estimated penetrance and credible intervals for the 15 most replicated copy number variants reported in schizophrenia patients from multiple studies. We also show a wider utility of CalPen by estimating penetrance using information on 145 SNPs significantly associated with schizophrenia.

## Materials and methods

### Description of the code

The python script developed is deposited in GitHub software development platform (https://github.com/dyex719/penetrance) and in the supporting files (S1 and S2 Files). Briefly, penetrance is calculated using population-based probabilistic method using the published dataset of the copy number variations (CNVs) at different locations in the genomes of schizophrenia patients and controls, wherein the data selection criteria were identical as described by Vassos et al. [3]. Based on the number of mutations identified in a given sample of controls and patients, median penetrance values were calculated from the formula:

$$P(D|G)=\frac{P(G|D)\cdot P(D)}{P(G|D)\cdot (PD)+P(G|\bar{D})\cdot P(\bar{D})}$$

Wherein *P*(*D*|*G*)] is the penetrance or the probability of developing the disease (*D*) for patients with genotype (*G*) carrying the CNV. *P*(*G*|*D*) is the frequency of the CNV in patients, *P*(*D)* is the lifetime morbid risk (baseline risk) for the disease, $P(G|\bar{D})$ is the frequency of the CNV in controls and $P(\bar{D})$ is the probability that an individual is normal (1 –*P*(*D*)).

For determination of the credible intervals, we first derived binomial prior distributions for controls and patients for each mutation by Wilson’s method [4] using Python ver. 3.5 and SciPy package ver. 1.2. These prior distributions were generated using the respective frequencies of mutations as the mean values for both cases and controls. For each prior distribution, the value corresponding to Mean + 2 σ gave the 97.5^th^ percentile value whereas Mean—2σ gave the 2.5^th^ percentile value (σ represents standard deviation). From the 2.5^th^ and 97.5^th^ percentiles of controls and patients, the upper bound and lower bounds of the credible intervals were calculated for each mutation by Bayesian method as follows

$$Lower\;Bound\;of\;credible\;interval=\frac{f_{2.5}(G|D)\cdot P(D)}{f_{2.5}(G|D)\cdot P(D)+f_{97.5}(G|\bar{D})\cdot P(\bar{D})}$$

$$Upper\;bound\;of\;credible\;interval=\frac{f_{97.5}(G|D)\cdot P(D)}{f_{97.5}(G|D)\cdot P(D)+f_{2.5}(G|\bar{D})\cdot P(\bar{D})}$$

Wherein *f*_*9*7.5_ and *f*_*2*.*5*_ correspond to the frequencies (proportions) at 97.5^th^ and 2.5^th^ percentiles respectively.

### Development of the web-based tool

The web application was created using Python Flask (a web application framework written in Python to develop websites; www.flask.pocoo.org) and PythonAnywhere (www.pythonanywhere.com; an online integrated development environment and web-hosting service based on the Python programming at www.python.org). HTML was used to allow the user to use a graphical user interface (GUI). Features were added so that this web-based HTML application alerts the user if any of the five valid inputs are not provided.

### Validation of CalPen

We used Pearson correlation coefficient as a means of validating the CalPen-derived penetrance values against those obtained in two published reports [3, 5]; (Table 1)]. For this purpose, the lifetime morbidity risk (baseline risk), the number of mutations and the sample sizes used were obtained from the reports and the values were entered in the dialog boxes of CalPen to obtain penetrance values and their credible intervals. Correlation coefficient for median penetrance was calculated and scatter plots were made using Microsoft excel 2016 [6]. In case of credible intervals, coefficient of range was calculated using the formula using values from CalPen and published reports

$$Coefficient\;of\;range\;of\;credible\;intervals=\frac{Upper\;bound\;value-Lower\;bound\;value}{Upper\;bound\;value+Lower\;bound\;value}$$

The individual values of the coefficients were then used to calculate the correlation coefficient and obtain scatter plots.

**Table 1:** Details of schizophrenia-associated copy number variants (CNVs) identified in different case-control studies. The reported penetrance values were used for comparison with the values obtained using CalPen. Credible intervals are shown in brackets. Data for CNVs numbered 1–8 were taken from Vassos *et al*. (2009; ref. 3) whereas from 9–21 were taken from Rosenfeld *et al*. (2011; ref. 5. del: deletion, dup: duplication.

S.No.	CNV	Number of cases	Number of Controls	Published values	CalPen values
				Penetrance (Credible Intervals)	Penetrance
1	1q21.1 del	7918	46502	0.061 (0.03–0.12)	0.061 (0.02–0.16)
2	2p16.3 del	2977	33746	0.020 (0.01–0.04)	0.021 (0.01–0.04)
3	15q11.2 del	7918	46497	0.020 (0.01–0.03)	0.020 (0.01–0.03)
4	15q13.3 del	7413	45103	0.074 (0.03–0.16)	0.074 (0.03–0.21)
5	16p13.1 dup	4816	37871	0.024 (0.01–0.04)	0.024 (0.01–0.05)
6	16p11.2 dup	8590	28406	0.069 (0.03–0.14)	0.069 (0.03–0.18)
7	17p12 del	5089	38884	0.067 (0.03–0.17)	0.067 (0.02–0.24)
8	22q11 del	7038	44602	0.553 (0.18–0.97)	0.553 (0.12–1.0)
9	Proximal 1q21.1 dup	48637	22246	0.173 (0.11–0.27)	0.165 (0.08–0.32)
10	Distal 1q21.1.del	33226	22246	0.369 (0.23–055)	0.346 (0.18–0.61)
11	Distal 1q21.1.dup	33226	22246	0.291 (0.17–0.47)	0.271 (0.13–0.53)
12	15q11.2 del	25113	22246	0.104 (0.08–0.13)	0.103 (0.08–0.14)
13	16p13.11 del	33226	22246	0.131 (0.79–0.21)	0.126 (0.06–0.26)
14	16p12.1 del	33226	22246	0.123 (0.79–0.19)	0.120 (0.06–0.23)
15	Distal 16p11.2 del	33226	22246	0.624 (0.27–0.94)	0.501 (0.18–0.93)
16	Distal 16p11.2 dup	33226	22246	0.112 (0.06–0.20)	0.108 (0.05–0.25)
17	Proximal 16p11.2 del	33226	22246	0.468 (0.31–0.64)	0.443 (0.26–0.69)
18	Proximal 16p11.2 dup	33226	22246	0.272 (0.17–0.41)	0.259 (0.14–0.47)
19	17q12 del	33226	22246	0.344 (0.14–0.70)	0.286 (0.09–0.73)
20	17q12 dup	33226	22246	0.211 (0.10–0.39)	0.194 (0.08–0.46)
21	22q11.21 dup	48637	22246	0.219 (0.15–0.32)	0.210 (0.12–0.37)

### Tests of significance

We used two methods to determine whether there is a significant difference in penetrance values reported and those obtained by CalPen. First, we used an online tool (https://www.socscistatistics.com/pvalues/pearsondistribution.aspx) and calculated the *P* value for obtaining the correlation coefficient (*r*) against n-2 degrees of freedom. As an independent approach, we also performed a χ2 test of association using the reported penetrance values as expected and Calpen values as observed values (https://www.graphpad.com/quickcalcs/chisquared1/?Format=C). A *P* value < 0.05 is taken as significant.

### Determination of penetrance values for CNVs and SNPs associated with schizophrenia

In order to obtain a more comprehensive estimates of penetrance for different CNVs found to be associated with schizophrenia (SZ), we identified a report suggesting 15 different CNVs that seem to have a better probability of getting replicated in future studies [7]; (Table 2). We then performed a thorough literature search and extracted the frequencies of these 15 CNVs from different case-control studies [8–14]. In addition, we also obtained frequencies from a different set of case-control studies that identified 145 SNPs associated with schizophrenia [15–29]; (Table 3). Of these, 128 were identified by the Psychiatric Genetics Consortium [29]. Using the sample sizes, the frequencies of these CNVs from these reports and the lifetime morbidity risk (baseline risk) of 0.7% [30], we calculated penetrance and the credible intervals.

**Table 2:** Published data on Schizophrenia–associated CNVs used for calculation of penetrance using CalPen. Del: Deletion; Dup: Duplication. Filled boxes indicate the identified CNV.

Reference	10	11	9	12	8	13	14
Controls	3,485	5,917	11,904	2,393	20,227	2,095	9,063
Cases	1,735	4,719	6,588	2,416	21,094	2,458	49
1q21.1 Del							
1q21.1 Dup							
2p16.3 NRXN1 Del							
3q29 Del							
7p36.3 VIPR2 WDR60 Del + Dup							
7q11.23 Dup							
8q22.2 VPS13B Del							
9p24.3 DMRT Del + Dup							
15q11.2 Del							
15q11.2 Dup							
15q13.3 Del							
16p11.2 proximal Dup							
16p11.2 distal Del							
16p13.11 Dup							
22q11.21 Del							

**Table 3:** Published data on Schizophrenia–associated SNPs used for calculation of penetrance using CalPen.

Details of the SNPs identified	Cases	Controls	Reference
rs175174	112	81	18
rs2270641	354	365	23
rs11743803	448	554	16
rs165599	398	440	20
rs947267	388	367	22
rs3738401	303	300	19
rs6603272	310	330	27
rs4938445	1,514	1,514	28
rs2373000	1,248	1,248	25
rs6280	685	768	28
rs1710921	1,112	1,135	26
rs115329265, rs1702294, rs11191419, rs2007044, rs4129585, rs35518360, chr7_2025096_I, rs4391122, rs2851447, chr2_200825237_I, rs4702, rs75968099, chr10_104957618_I, rs12887734, chr10_104957618_I, rs8042374, rs13240464, rs10791097, rs11693094, rs1378559, rs7893279, rs12826178, rs12129573, rs6704768, rs55661361, rs9636107, chr11_46350213_D, rs7907645, chr3_180594593_I, rs6065094, rs11682175, rs950169, rs72934570, rs6434928, rs9607782, rs36068923, rs17194490, rs2514218, rs75059851, rs2535627, rs12691307, chr22_39987017_D, rs7432375, chr18_52749216_D, rs111294930, rs2973155, rs5937157, rs4523957, rs12704290, rs12903146, rs11210892, rs2905426, rs140505938, chr6_84280274_D, rs4648845, rs7405404, rs6466055, chr1_8424984_D, rs4766428, rs10520163, rs117074560, rs6002655, chr2_146436222_I, rs9420, rs11027857, rs1498232, rs3735025, rs11139497, rs77149735, rs56205728, rs2053079, rs16867576, rs4330281, rs3849046, rs2693698, rs2332700, rs1501357, rs6984242, chr1_243881945_I, rs79212538, rs3768644, rs77502336, rs6704641, rs59979824, rs1106568, rs10503253, rs10043984, rs11685299, rs7819570, rs715170, rs9922678, rs78322266, rs2068012, rs832187, rs8044995, chr2_149429178_D, rs8082590, rs12148337, rs12325245, rs2239063, rs12522290, rs10803138, rs73229090, rs324017, rs12845396, rs55833108, rs9841616, rs76869799, rs1339227, chr7_24747494_D, rs4388249, rs215411, rs11740474, rs1023500, rs12421382, rs211829, rs679087, rs75575209, rs7801375, rs14403, rs6670165, rs7523273, rs7267348, rs4240748, rs2909457, rs56873913, rs190065944, rs10860964, chr5_140143664_I	36,989	113,075	29
rs1800532	572	1049	17
rs1006737	552	1132	15
rs1801028	420	403	24
Total	45,405	122,761	

## Results and discussion

**Fig 1:**
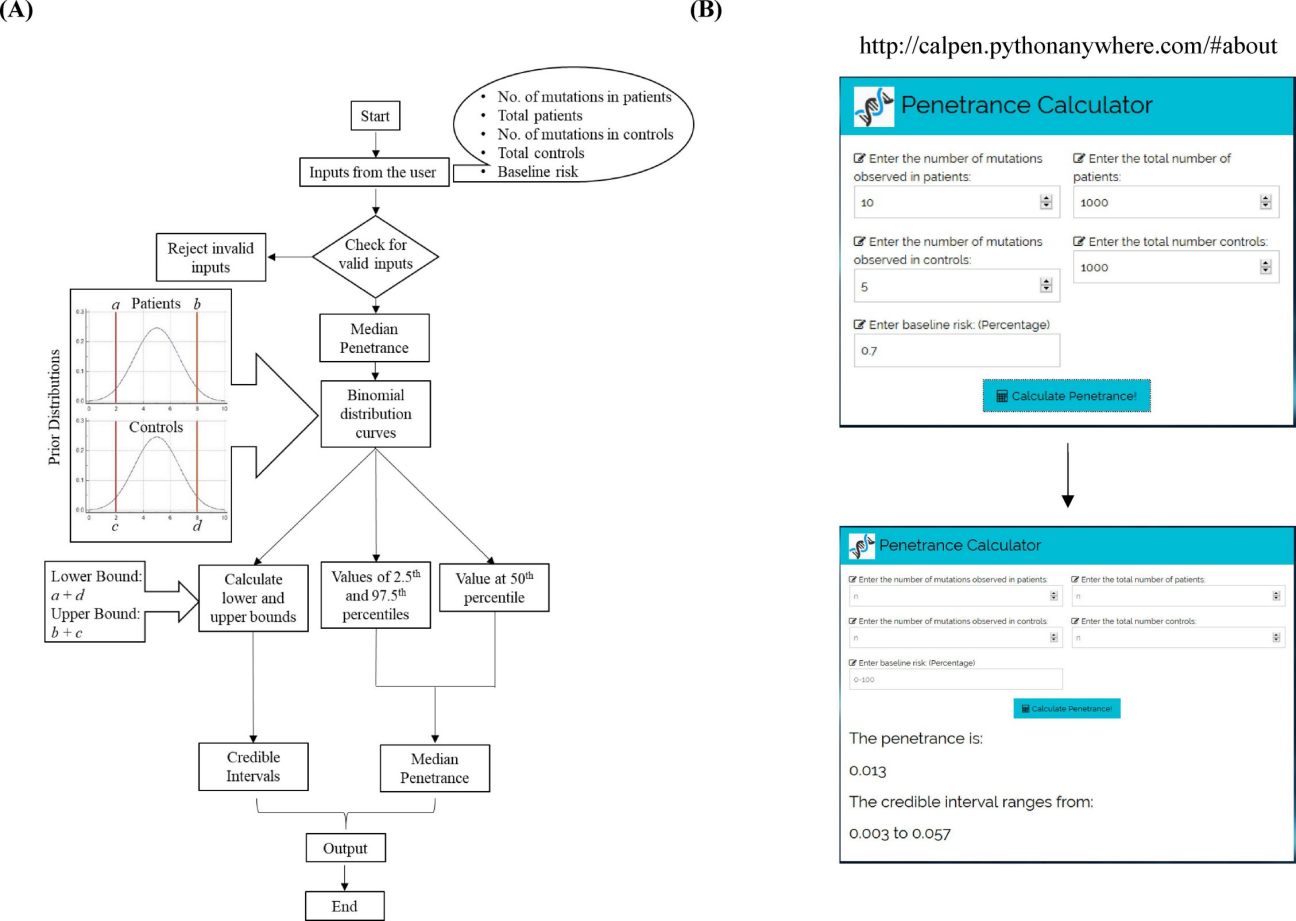
Development of CalPen, a web-based tool and its evaluation in calculating penetrance for copy number variants associated with schizophrenia. (A) Schematic drawing of the pipeline used to determine the median penetrance and the critical intervals. (B) Screen shots showing the process of calculation of penetrance and credible intervals for a mutation (CNV). The numbers in the dialog boxes are indicative.

In order to develop a web-based tool for calculation of penetrance (CalPen), we used the same parameters as described by Vassos *et al*. [3] and Python script in the SciPy package (see Materials and Methods). A schematic of the workflow resulting in computation of median penetrance and credible intervals is shown in Fig 1A. An example of steps involved in using CalPen is shown in Fig 1B wherein the user needs to enter the appropriate number against the dialog boxes given. For example, the data in Fig 1B indicates that there are 10 mutations in 1000 patients but five among 1000 controls. After entering the baseline risk (lifetime morbidity risk), the user needs to click the dialog box named ‘Calculate Penetrance’ and, the software gets forwarded to the next webpage showing the values of penetrance and credible intervals at the bottom. The user does not need to go to the previous webpage to start with another mutation but can continue from the same page by entering a new set of relevant numbers.

**Fig 2:**
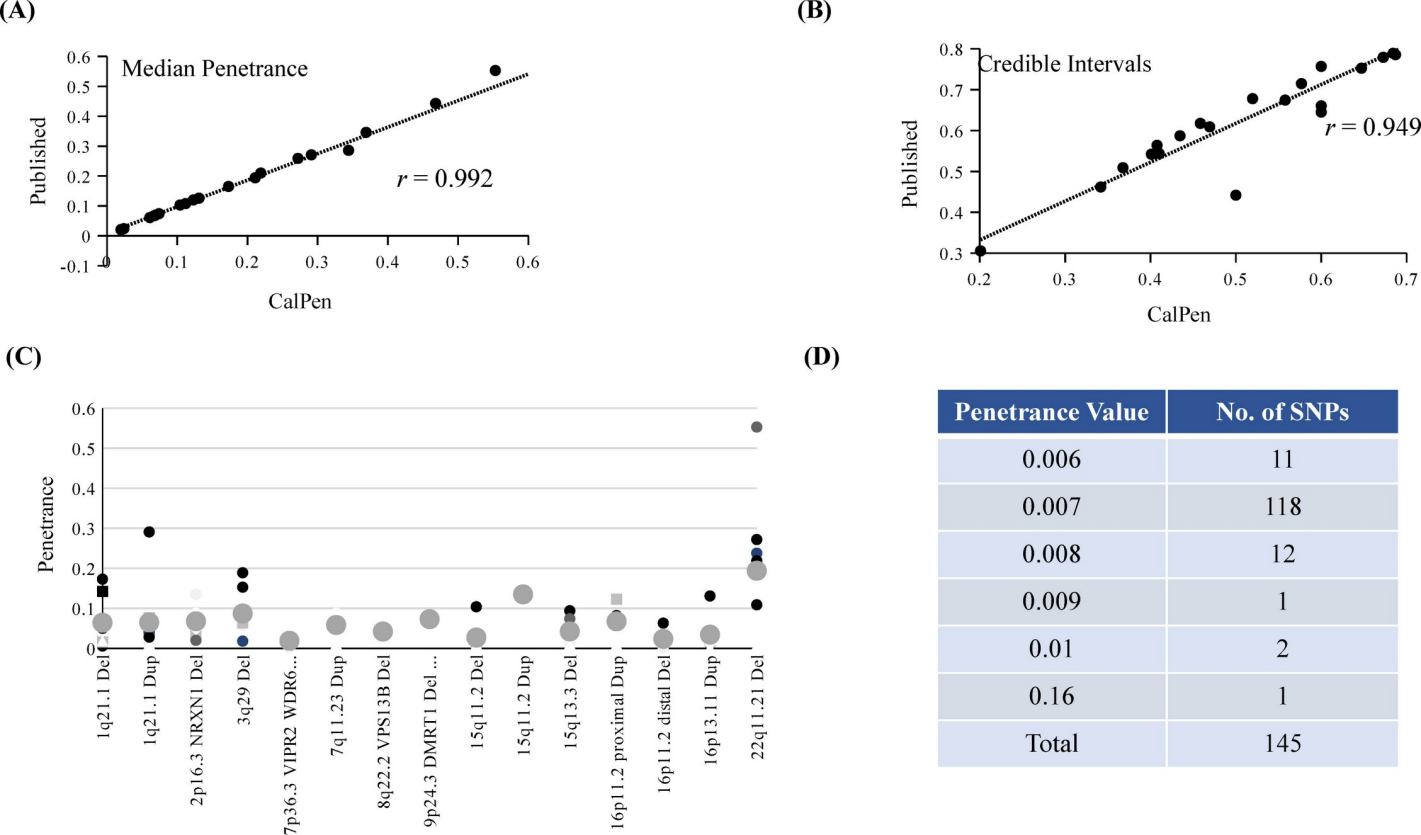
CalPen-mediated determination of penetrance values and credible intervals for different CNVs. (A) Validation of penetrance values obtained using CalPen by correlation with those obtained from published reports using the same data sets. (B) Correlation of credible intervals for the data shown in (Fig 2A). (C) Estimates of penetrance values for different CNVs found to be associated with schizophrenia from multiple studies. For each CNV on the X-axis, values from multiple studies are plotted on the Y axis. Average value is shown as a larger red circle. (D) Penetrance value distribution among 145 SNPs associated with schizophrenia.

In order to validate the performance of CalPen, we used the published data from Vassos *et al* [3] and Rosenfeld *et al* [5]. In both cases, we used baseline risks as given by the authors. Data from CalPen and the two published reports is shown in Table 1. A comparison of the median penetrance values obtained using CalPen with the reported values gave a coefficient of correlation (*r*) of 0.992 (Fig 2A), with a *P* value < 0.001 indicating a significant degree of association between reported and CalPen-derived penetrance data. As an independent measure we used χ2 test with Yate’s correction, which gave a value of 0.04, corresponding to a *P* value of 1.0, indicating a high-degree of agreement in the two sets of values. In case of credible intervals, we first calculated the coefficient of range of these intervals and then used for calculation of correlation coefficient. The data shown in Fig 2B gave a *r* value of ~0.95, again indicating that there is a significant similarity between the published and CalPen-calculated credible intervals (*P* <0.001). As in case of penetrance values, a χ2 test with Yates correction gave a *P* value of 1.0 indicating that the published credible intervals were very similar to those calculated using CalPen. Taken together, these results suggest that CalPen software gives reliable values of both penetrance and credible intervals.

To demonstrate the wider utility of CalPen, we chose schizophrenia (SZ) as an example of complex disorder in which two categories of variants *viz*., Copy Number Variants (CNVs) and Single nucleotide polymorphisms (SNPs) are widely studied. CNVs are sub-microscopic deletions and duplications ranging in size from few kilobases to a few megabases, affecting one to many genes, constituting about 5–10% of human variation [31]. Among the different CNVs identified in SZ, meta-analysis resulted in identification of a specific set of 15 CNVs that are more likely to be replicated in a diverse set of populations [7]. Data on these 15 CNVs was obtained from different published reports [8–14]; (Table 2) and penetrance values were calculated using CalPen (Fig 2C). The data suggests that for a given CNV, there was a range of penetrance values from different reports. For example, 3q29 deletions showed penetrance values ranging from 1.8% to 15.3%. Overall the average penetrance of the 15 CNVs is ~7%, meaning that among 100 individuals with a CNV, there is a likelihood of seven individuals being abnormal. CNVs of 22q11.21 appear to have the highest average penetrance (~20%) whereas 15q11.2 deletions, which also represent variants of uncertain significance, have the lowest average penetrance (~ 1.4%). We also estimated the penetrance values of 128 SNPs using a large set of data reported by the psychiatric Genetics Consortium [29] and other reports that identified 17 SNPs among schizophrenia patients [15–28]; (Table 3). In contrast to CNVs, the odds ratios of the SNPs are always lower, rarely approach a ratio of 1.5 [32] and therefore are likely to have lesser penetrance than CNVs. In agreement with this expectation, 117 out of 145 SNPs studied showed a penetrance of 0.7%. Eleven SNPs showed lowest (0.6%) and rs1801028 showed the highest (1.6%) penetrance values (Fig 2D; S1 Table).

## Conclusion

In conclusion, CalPen is a straight forward tool for accurate determination of penetrance and credible intervals of mutations/variants associated with complex disorders and circumvents the bottleneck of the requirement of programming skills. At this juncture, this tool can calculate penetrance for one variant at a time and does not allow a set of variants identified in case-control studies to be analyzed together. Also, it is not possible to perform complex calculations resulting in estimations of combined penetrance in patients with more than one variant. With these improvements, CalPen in future may enable in better understanding of the phenotypic outcomes in complex disease genetics. For routine penetrance calculations, this web-based tool can be accessed from http://calpen.pythonanywhere.com/#about.

## Supporting information

S1 Table Penetrance value distribution of 145 SNPs associated with schizophrenia. (PDF)

S1 File Python source code. (PDF)

S2 File HTML source code. (PDF)
