# Correction: CalPen (Calculator of Penetrance), a web-based tool to estimate penetrance in complex genetic disorders

**DOI:** 10.1371/journal.pone.0235547

**Published:** 2020-06-25

**Authors:** Aditya Addepalli, Sakhare Kalyani, Minali Singh, Debashree Bandyopadhyay, K. Naga Mohan

The fifth author's initials appear incorrectly in the citation. The correct citation is: Addepalli A, Kalyani S, Singh M, Bandyopadhyay D, Mohan KN (2020) CalPen (Calculator of Penetrance), a web-based tool to estimate penetrance in complex genetic disorders. PLoS ONE 15(1): e0228156. https://doi.org/10.1371/journal.pone.0228156

There is an error in the affiliation for all authors. The correct affiliation is: Department of Biological Sciences, BITS Pilani Hyderabad Campus, Hyderabad, Telangana, India
