# Supplementary material for: CalPen (Calculator of Penetrance), a web-based tool to estimate penetrance in complex genetic disorders
Source: PLoS One. 2020 Jan 29;15(1):e0228156. doi: 10.1371/journal.pone.0228156 (PMC6988981; doi:10.1371/journal.pone.0228156)
Supplement: S1 Table — (PDF) [file pone.0228156.s001.pdf]

Supplementary Material 3

Table. Penetrance value distribution of 145 SNPs associated with schizophrenia.

SNP	Penetrance value
rs1800532<br>rs2373000<br>rs1710921<br>rs1702294<br>chr10_104957618_I<br>rs12826178<br>rs72934570<br>rs12704290<br>rs140505938<br>rs117074560<br>rs73229090	0.006
rs3738401<br>rs3738401<br>rs165599<br>rs4938445<br>rs2270641<br>rs6280<br>rs115329265<br>rs11191419<br>rs2007044<br>rs4129585<br>rs35518360<br>chr7_2025096_I<br>rs4391122<br>rs2851447<br>chr2_200825237_I<br>rs4702<br>rs75968099<br>rs12887734<br>rs8042374<br>rs13240464<br>rs10791097<br>rs11693094<br>rs1378559<br>rs7893279<br>rs12129573<br>rs6704768<br>rs55661361	0.007

rs9636107<br>chr11_46350213_D<br>rs7907645<br>chr3_180594593_I<br>rs6065094<br>rs11682175<br>rs950169<br>rs6434928<br>rs36068923<br>rs2514218<br>rs75059851<br>rs2535627<br>rs12691307<br>chr22_39987017_D<br>rs7432375<br>chr18_52749216_D<br>rs111294930<br>rs2973155<br>rs5937157<br>rs4523957<br>rs12903146<br>rs11210892<br>rs2905426<br>chr6_84280274_D<br>rs4648845<br>rs7405404<br>rs6466055<br>chr1_8424984_D<br>rs4766428<br>rs10520163<br>rs6002655<br>rs9420<br>rs11027857<br>rs1498232<br>rs3735025<br>rs11139497<br>rs56205728<br>rs2053079<br>rs16867576<br>rs4330281<br>rs3849046<br>rs2693698<br>rs2332700<br>rs1501357<br>rs6984242<br>chr1_243881945_I	

rs3768644<br>rs77502336<br>rs6704641<br>rs59979824<br>rs1106568<br>rs10503253<br>rs10043984<br>rs11685299<br>rs715170<br>rs9922678<br>rs2068012<br>rs832187<br>rs8044995<br>chr2_149429178_D<br>rs8082590<br>rs12148337<br>rs12325245<br>rs2239063<br>rs12522290<br>rs10803138<br>rs324017<br>rs12845396<br>rs55833108<br>rs9841616<br>rs76869799<br>rs1339227<br>rs4388249<br>rs215411<br>rs11740474<br>rs1023500<br>rs12421382<br>rs211829<br>rs679087<br>rs75575209<br>rs7801375<br>rs14403<br>rs6670165<br>rs7523273<br>rs7267348<br>rs4240748<br>rs2909457<br>rs56873913<br>rs190065944<br>rs10860964<br>chr5_140143664_I	
rs1006737	0.008

rs175174<br>rs165599<br>rs1801028<br>rs9607782<br>rs17194490<br>chr2_146436222_I<br>rs77149735<br>rs79212538<br>rs7819570<br>rs78322266<br>chr7_24747494_D	
rs947267	0.009
rs11743803<br>rs6603272	0.01
rs1801028	0.016
