# Supplementary material for: CalPen (Calculator of Penetrance), a web-based tool to estimate penetrance in complex genetic disorders
Source: PLoS One. 2020 Jan 29;15(1):e0228156. doi: 10.1371/journal.pone.0228156 (PMC6988981; doi:10.1371/journal.pone.0228156)
Supplement: S1 File — (PDF) [file pone.0228156.s002.pdf]

```
from flask import Flask, render_template,request
from scipy.stats import beta
import scipy.stats as st
import numpy as np
from scipy import stats, optimize
from sys import float_info

app = Flask(__name__)
app.config["DEBUG"] = True

comments = [0,0,0]

def mean_confidence_interval(data, confidence=0.95):
    return st.t.interval(confidence, len(data)-1, loc=np.mean(data), scale=st.sem(data))

def Bayesian(prob_cases,prob_controls, baseline_risk):
    return (prob_cases*baseline_risk) /
((prob_cases*baseline_risk)+(prob_controls*(1-(baseline_risk))))

def proportion_confint(count, nobs, alpha=0.05, method='normal'):
    q_ = count * 1. / nobs
    alpha_2 = 0.5 * alpha
    if method == 'normal':
        std_ = np.sqrt(q_ * (1 - q_) / nobs)
        dist = stats.norm.isf(alpha / 2.) * std_
        ci_low = q_ - dist
        ci_upp = q_ + dist
    elif method == 'binom_test':
        def func(qi):
            return stats.binom_test(q_ * nobs, nobs, p=qi) - alpha
        if count == 0:
            ci_low = 0
        else:
            ci_low = optimize.brentq(func, float_info.min, q_)
        if count == nobs:
            ci_upp = 1
        else:
            ci_upp = optimize.brentq(func, q_, 1. - float_info.epsilon)
    elif method == 'beta':
        ci_low = stats.beta.ppf(alpha_2, count, nobs - count + 1)
        ci_upp = stats.beta.isf(alpha_2, count + 1, nobs - count)
    elif method == 'agresti_coull':
        crit = stats.norm.isf(alpha / 2.)
```

```
        nobs_c = nobs + crit**2
        q_c = (count + crit**2 / 2.) / nobs_c
        std_c = np.sqrt(q_c * (1. - q_c) / nobs_c)
        dist = crit * std_c
        ci_low = q_c - dist
        ci_upp = q_c + dist
    elif method == 'wilson':
        crit = stats.norm.isf(alpha / 2.)
        crit2 = crit**2
        denom = 1 + crit2 / nobs
        center = (q_ + crit2 / (2 * nobs)) / denom
        dist = crit * np.sqrt(q_ * (1. - q_) / nobs + crit2 / (4. * nobs**2))
        dist /= denom
        ci_low = center - dist
        ci_upp = center + di        # inverting the binomial test
st
    # method adjusted to be more forgiving of misspellings or incorrect option name
    elif method[:4] == 'jeff':
        ci_low, ci_upp = stats.beta.interval(1 - alpha, count + 0.5,
                                             nobs - count + 0.5)
    else:
        raise NotImplementedError('method "%s" is not available' % method)
    return (ci_low, ci_upp)

def probabilities(a,b,quantiles=0.500):
    candidates = []
    # Define the Beta distibution
    # Increase this value to increase the precision
    x = np.linspace(beta.ppf(0.001, a, b),beta.ppf(0.999, a, b), 10000)
    data_points = (np.asarray(beta.cdf(x, a, b)))
    for i,j in enumerate((range(len(data_points)))):
        if(round(data_points[j],3) == quantiles):
            # print(data_points[j])
            candidates.append(data_points[j])
    # Look for the value closest to 0.5 in the cdf of the beta distribution
    closest_value = min(candidates, key=lambda x:abs(x-quantiles))
    # print(closest_value)
    return (beta.ppf(closest_value,a,b))

def penetrance_confint(ac_case, n_case, ac_control, n_control, baseline_risk):
    case_confint =  proportion_confint(count=ac_case,nobs=2*n_case,method='wilson')
    control_confint =  proportion_confint(count=ac_control,nobs=2*n_control,method='wilson')
    lower_bound = Bayesian(case_confint[0],control_confint[1],baseline_risk)
```

```
    upper_bound = Bayesian(case_confint[1],control_confint[0],baseline_risk)
    return (lower_bound,upper_bound)

@app.route('/')
def my_form():
    return render_template('main_page.html')

@app.route('/', methods=['POST'])
def my_form_post():
    text = request.form['text']
    text1 = request.form['text1']
    text2 = request.form['text2']
    text3 = request.form['text3']
    text4 = request.form['text4']
    # Sample Data: 22q11 del from Kirov's data.
    x = 1
    y = 1
    # Includes the hidden case factor of unscreened controls developing schizophrenia.
    n = int(text)
    N = int(text1)
    m = int(text2)
    M = int(text3)
    P = float(text4)
    P = P/100
    L = 1
    n = n + M*(1-L)*P*n/N
    N = N + M*(1-L)*P
    m = max(0, m - M*(1-L)*P*n/N)
    M =  M - M*(1-L)*P
    # For cases
    a = x + n
    b = y + N - n
    # For controls
    A = x + m
    B = y + M - m
    penetrance_5 = Bayesian(probabilities(a,b,0.500),probabilities(A,B,0.500),P)
    penetrance_25 = Bayesian(probabilities(a,b,0.025),probabilities(A,B,0.025),P)
    penetrance_975 = Bayesian(probabilities(a,b,0.975),probabilities(A,B,0.975),P)
    median_penetrance = np.median([penetrance_5,penetrance_25,penetrance_975])
    output = round(median_penetrance,3)
    comments[0] = output
    lower,upper = penetrance_confint(n,N,m,M,P)
    comments[1] = round(lower,3)
```

```
comments[2] = round(upper,3)
return render_template("main_page.html", comments= comments)
```
