# Supplementary material for: CalPen (Calculator of Penetrance), a web-based tool to estimate penetrance in complex genetic disorders
Source: PLoS One. 2020 Jan 29;15(1):e0228156. doi: 10.1371/journal.pone.0228156 (PMC6988981; doi:10.1371/journal.pone.0228156)
Supplement: S2 File — (PDF) [file pone.0228156.s003.pdf]

```html
<!DOCTYPE html>
<html>
  <title>CalPen</title>
  <meta name="viewport" content="width=device-width, initial-scale=1">
  <link rel="stylesheet" href="https://www.w3schools.com/w3css/4/w3.css">
  <link rel="stylesheet" href="https://fonts.googleapis.com/css?family=Raleway">
  <link rel="stylesheet"
href="https://cdnjs.cloudflare.com/ajax/libs/font-awesome/4.7.0/css/font-awesome.min.css">
  <style>
    body,h1,h2,h3,h4,h5,h6 {font-family: "Raleway", Arial, Helvetica, sans-serif}
  </style>
  <body class="w3-light-grey">
    <!-- Navigation Bar -->
    <div class="w3-bar w3-white w3-large">
      <a href="/" class="w3-bar-item w3-button w3-cyan w3-mobile"><img
src="/static/dnaicon.png" alt="Icon" style="width:25px;height:25px;"></i>CalPen</a>
      <a href="#getting-started" class="w3-bar-item w3-button w3-mobile
w3-hover-text-cyan">Getting Started</a>
      <a href="#about" class="w3-bar-item w3-button w3-mobile
w3-hover-text-cyan">About</a>
    </div>
    <!-- Header -->
    <header class="w3-display-container w3-content" style="max-width:2000px;">
      <img class="w3-image" src="/static/dnaabstract.png" alt="Background"
style="min-width:1000px" width="2500" height="1500">
      <div class="w3-display-middle w3-padding w3-col l6 m8">
        <div class="w3-container w3-cyan">
          <h2><img src="/static/dnaicon.png" alt="Icon" style="width:50px;height:50px;">
Penetrance Calculator</h2>
        </div>
        <div class="w3-container w3-white w3-padding-16">
          <form method="POST">
            <div class="w3-row-padding" style="margin:0 -16px;">
              <div class="w3-half w3-margin-bottom">
                <label><i class="fa fa-edit"></i> Enter the number of mutations observed in
patients:</label>
                <input class="w3-input w3-border" type="number" placeholder="8" name="text"
min="0" required>
              </div>
              <div class="w3-half">
                <label><i class="fa fa-edit"></i> Enter the total number of patients:</label>
                <input class="w3-input w3-border" type="number" placeholder="5089"
name="text1" min="0" required>
```

```
                    </div>
                  </div>
                  <div class="w3-row-padding" style="margin:0 -16px;">
                    <div class="w3-half w3-margin-bottom">
                      <label><i class=<!DOCTYPE html>
<html>
<title>CalPen</title>
<meta name="viewport" content="width=device-width, initial-scale=1">
<link rel="stylesheet" href="https://www.w3schools.com/w3css/4/w3.css">
<link rel="stylesheet" href="https://fonts.googleapis.com/css?family=Raleway">
<link rel="stylesheet"
href="https://cdnjs.cloudflare.com/ajax/libs/font-awesome/4.7.0/css/font-awesome.min.css">
<style>
body,h1,h2,h3,h4,h5,h6 {font-family: "Raleway", Arial, Helvetica, sans-serif}
</style>
<body class="w3-light-grey">

<!-- Navigation Bar -->
<div class="w3-bar w3-white w3-large">
  <a href="/" class="w3-bar-item w3-button w3-cyan w3-mobile"><img src="/static/dnaicon.png"
alt="Icon" style="width:25px;height:25px;"></i>CalPen</a>
  <a href="#getting-started" class="w3-bar-item w3-button w3-mobile
w3-hover-text-cyan">Getting Started</a>
  <a href="#about" class="w3-bar-item w3-button w3-mobile w3-hover-text-cyan">About</a>
</div>
<!-- Header -->
<header class="w3-display-container w3-content" style="max-width:2000px;">
  <img class="w3-image" src="/static/dnaabstract.png" alt="Background"
style="min-width:1000px" width="2500" height="1500">
  <div class="w3-display-middle w3-padding w3-col l6 m8">
    <div class="w3-container w3-cyan">
      <h2><img src="/static/dnaicon.png" alt="Icon" style="width:50px;height:50px;"> Penetrance
Calculator</h2>
    </div>
    <div class="w3-container w3-white w3-padding-16">
      <form method="POST">
        <div class="w3-row-padding" style="margin:0 -16px;">
          <div class="w3-half w3-margin-bottom">
            <label><i class="fa fa-edit"></i> Enter the number of mutations observed in
patients:</label>
            <input class="w3-input w3-border" type="number" placeholder="8" name="text" min="0"
required>
          </div>
```

```
          <div class="w3-half">
            <label><i class="fa fa-edit"></i> Enter the total number of patients:</label>
            <input class="w3-input w3-border" type="number" placeholder="5089" name="text1"
min="0" required>
          </div>
        </div>

        <div class="w3-row-padding" style="margin:0 -16px;">
          <div class="w3-half w3-margin-bottom">
            <label><i class="fa fa-edit"></i> Enter the number of mutations observed in
controls:</label>
            <input class="w3-input w3-border" type="number" placeholder="6" name="text2"
min="0" required>
          </div>
          <div class="w3-half">
            <label><i class="fa fa-edit"></i> Enter the total number controls:</label>
            <input class="w3-input w3-border" type="number" placeholder="38884" name="text3"
min="0" required>
          </div>
        </div>

        <div class="w3-row-padding" style="margin:0 -16px;">
          <div class="w3-half w3-margin-bottom">
            <label><i class="fa fa-edit"></i> Enter baseline risk: (Percentage)</label>
            <input class="w3-input w3-border" type="text" placeholder="0.72" name="text4" min="0"
required>
          </div>
        </div>
        <center><button class="w3-button w3-cyan" type="submit"><i class="fa fa-calculator"></i>
Calculate Penetrance!</button></center>
        {% if comments %}
        <p><h2> The penetrance is: </h2></p> <h2> {{comments[0]}}</h2>
        <p><h2> The confidence interval ranges from: </h2></p> <p><h2>{{comments[1]}} to
{{comments[2]}} </h2></p>
        {% endif %}
      </form>
    </div>
  </div>
</header>

<div class="w3-row-padding">
    <div class="w3-col l4 m7" align="left" id="getting-started">
      <h3>Getting Started</h3>
```

<h6>

The following is an illustration of how the application works:

<p>
In order to calculate penetrance for a mutation, five types of data are needed:

The number of mutations identified in a patient sample

The number of patients studied

The number of mutations identified in the control sample

The number of controls studied

The general incidence of the disease under investigation in the population from which patients and controls are sampled.

</p>

<p>
Using these values, a Bayesian probabilistic method is employed to calculate penetrance. This involves simulation using the Python Scipy package and extraction of 2.5, 50 and 97.5% quantiles to obtain the median penetrance, and its ~95% credible intervals.
</p>

<p>
An example of this process is given as default values in the above fields.
</p>
<p>
This method is described in more detail in the papers written by Vassos et al.

E. Vassos, D. A. Collier, S. Holden, C. Patch, D. Rujescu, D. St Clair, and C. M. Lewis, “Penetrance for copy number variants associated with schizophrenia,” Human molecular genetics, vol. 19, no. 17, pp. 3477–3481, 2010.

G. Kirov, E. Rees, J. T. Walters, V. Escott-Price, L. Georgieva, A. L. Richards, K. D. Chambert, G. Davies, S. E. Legge, J. L. Moran, et al., “The penetrance of copy number variations for schizophrenia and developmental delay,” Biological psychiatry, vol. 75, no. 5, pp. 378–385, 2014.

```
    </ol>
</p>



      </h6>
    </div>
        <div class="w3-col l8 m5" align="right;" id="about">
      <h3>About</h3>
      <h6>


<p>KNM lab is supported by OPERA award from BITS Pilani and by the Centre for Human Diseases.</p>
<p>AA developed the software which was analysed by KNM and DB.</p>


      </h6>
      </div>
</div>

<footer class="w3-padding-32 w3-black w3-center w3-margin-top">
  <h5>Source Code Available at <a href="https://github.com/Dyex719/Penetrance" class="w3-hover-text-cyan">Github <i class="fa fa-github"></i></a></h5>
  <p>Powered by <a href="http://flask.pocoo.org/" target="_blank" class="w3-hover-text-cyan">Flask</a> and <a href="https://www.w3schools.com/w3css/default.asp" target="_blank" class="w3-hover-text-cyan">w3.css</a></p>
  <p>Hosted by <a href="https://www.pythonanywhere.com/" target="_blank" class="w3-hover-text-cyan">PythonAnywhere</a></p>
</footer>
</body>
</html>
"fa fa-edit"></i> Enter the number of mutations observed in controls:</label>
                        <input class="w3-input w3-border" type="number" placeholder="6" name="text2" min="0" required>
                      </div>
                      <div class="w3-half">
                        <label><i class="fa fa-edit"></i> Enter the total number controls:</label>
                        <input class="w3-input w3-border" type="number" placeholder="38884" name="text3" min="0" required>
                      </div>
                    </div>
```

```
            <div class="w3-row-padding" style="margin:0 -16px;">
              <div class="w3-half w3-margin-bottom">
                <label><i class="fa fa-edit"></i> Enter baseline risk: (Percentage)</label>
                <input class="w3-input w3-border" type="text" placeholder="0.72" name="text4"
min="0" required>
              </div>
            </div>
            <center><button class="w3-button w3-cyan" type="submit"><i class="fa
fa-calculator"></i> Calculate Penetrance!</button></center>
            {% if comments %}
            <p>
            <h2> The penetrance is: </h2>
            </p>
            <h2> {{comments[0]}}</h2>
            <p>
            <h2> The confidence interval ranges from: </h2>
            </p>
            <p>
            <h2>{{comments[1]}} to {{comments[2]}} </h2>
            </p>
            {% endif %}
          </form>
        </div>
      </div>
    </header>
    <div class="w3-row-padding">
      <div class="w3-col l4 m7" align="left" id="getting-started">
        <h3>Getting Started</h3>
        <h6>
          The following is an illustration of how the application works:
          <p>
            In order to calculate penetrance for a mutation, five types of data are needed:
          <ol>
            <li>The number of mutations identified in a patient sample</li>
            <li>The number of patients studied</li>
            <li>The number of mutations identified in the control sample</li>
            <li>The number of controls studied</li>
            <li>The general incidence of the disease under investigation in the population from
which patients and controls are sampled.</li>
          </ol>
          </p>
          <p>
```

```
                  Using these values, a Bayesian probabilistic method is employed to calculate
penetrance. This involves simulation using the Python Scipy package and extraction of 2.5, 50
and 97.5% quantiles to obtain the median penetrance, and its ~95% credible intervals.
                </p>
                <p>
                  An example of this process is given as default values in the above fields.
                </p>
                <p>
                  This method is described in more detail in the papers written by Vassos et al.</a>
                <ol>
                  <li>E. Vassos, D. A. Collier, S. Holden, C. Patch, D. Rujescu, D. St Clair, and C. M.
Lewis,
                    “Penetrance for copy number variants associated with schizophrenia,” Human
molecular
                    genetics, vol. 19, no. 17, pp. 3477–3481, 2010.
                  </li>
                  <li>G. Kirov, E. Rees, J. T. Walters, V. Escott-Price, L. Georgieva, A. L. Richards, K.
D.
                    Chambert, G. Davies, S. E. Legge, J. L. Moran, et al., “The penetrance of copy
number
                    variations for schizophrenia and developmental delay,” Biological psychiatry, vol.
75, no. 5,
                    pp. 378–385, 2014.
                  </li>
                </ol>
                </p>
              </h6>
            </div>
            <div class="w3-col l8 m5" align="right;" id="about">
              <h3>About</h3>
              <h6>
                <p>KNM lab is supported by OPERA award from BITS Pilani and by the Centre for
Human Diseases.</p>
                <p>AA developed the software which was analysed by KNM and DB.</p>
              </h6>
            </div>
          </div>
          <footer class="w3-padding-32 w3-black w3-center w3-margin-top">
            <h5>Source Code Available at <a href="https://github.com/Dyex719/Penetrance"
class="w3-hover-text-cyan">Github <i class="fa fa-github"></i></a></h5>
            <p>Powered by <a href="http://flask.pocoo.org/" target="_blank"
class="w3-hover-text-cyan">Flask</a> and <a
```

```
href="https://www.w3schools.com/w3css/default.asp" target="_blank"
class="w3-hover-text-cyan">w3.css</a></p>
        <p>Hosted by <a href="https://www.pythonanywhere.com/" target="_blank"
class="w3-hover-text-cyan">PythonAnywhere</a></p>
      </footer>
   </body>
</html>
```
